# The Immunomodulatory Effect of Triptolide on Mesenchymal Stromal Cells

**DOI:** 10.3389/fimmu.2021.686356

**Published:** 2021-08-16

**Authors:** Haiping He, Atsuko Takahashi, Takeo Mukai, Akiko Hori, Miwako Narita, Arinobu Tojo, Tonghua Yang, Tokiko Nagamura-Inoue

**Affiliations:** ^1^Department of Cell Processing and Transfusion, The Institute of Medical Science, The University of Tokyo, Minato-ku, Japan; ^2^Department of Hematology, The First People’s Hospital of Yunnan Province, The Affiliated Hospital of Kunming University of Science and Technology, Kunming, China; ^3^Laboratory of Hematology and Oncology, School of Health Science, Niigata University Faculty of Medicine, Niigata, Japan; ^4^Division of Molecular Therapy, Center for Advanced Medical Research, Institute of Medical Science, The University of Tokyo, Minato-ku, Japan

**Keywords:** mesenchymal stromal cells, umbilical cord, triptolide, immunosuppression, PD-L1

## Abstract

Mesenchymal stromal cells (MSCs) are known to have immunosuppressive ability and have been used in clinical treatment of acute graft-*versus*-host disease, one of severe complications of the hematopoietic stem cell transplantation. However, MSCs are activated to suppress the immune system only after encountering an inflammatory stimulation. Thus, it will be ideal if MSCs are primed to be activated and ready to suppress the immune reaction before being administered. Triptolide (TPL) is a diterpene triepoxide purified from a Chinese herb-Tripterygium wilfordii Hook.f. It has been shown to possess anti-inflammatory and immunosuppressive properties *in vitro*. In this study, we aimed to use TPL to prime umbilical cord-derived MSCs (TPL-primed UC-MSCs) to enter a stronger immunosuppressive status. UC-MSCs were primed with TPL, which was washed out thoroughly, and the TPL-primed UC-MSCs were resuspended in fresh medium. Although TPL inhibited the proliferation of UC-MSCs, 0.01 μM TPL for 24 h was tolerable. The surface markers of TPL-primed UC-MSCs were identical to those of non-primed UC-MSCs. TPL-primed UC-MSCs exhibited stronger anti-proliferative effect for activated CD4+ and CD8+ T cells in the allogeneic mixed lymphocyte reaction assay than the non-primed UC-MSCs. TPL-primed UC-MSCs promoted the expression of IDO-1 in the presence of IFN-γ, but TPL alone was not sufficient. Furthermore, TPL-primed UC-MSCs showed increased expression of PD-L1. Conclusively, upregulation of IDO-1 in the presence of IFN-γ and induction of PD-L1 enhances the immunosuppressive potency of TPL-primed UC-MSCs on the proliferation of activated T cells. Thus, TPL- primed MSCs may provide a novel immunosuppressive cell therapy.

## Impact Statement (85 words)

The study focuses on the immunosuppressive properties of the mesenchymal stromal cells (MSCs) which has already found application in clinical settings for the treatment of acute graft-*versus*-host disease (GVHD), a severe complication of the hematopoietic stem cell transplantation (HSCT). The disadvantage though, is that MSCs get activated only after encountering an inflammatory stimulation. The authors provide evidence that pre-treatment of UC-MSCs with TPL, the active component of a Chinese herb, primes MSCs to be activated and ready to suppress the immune reaction before being administered.

## Introduction

Mesenchymal stromal cells (MSCs) are known to have immunosuppressive abilities and have been used clinically to treat the acute graft-*versus*-host disease (GVHD), which accounts as one of the most severe complications after hematopoietic stem cell transplantation (HSCT) (1). However, MSCs are activated to suppress the immune system only upon their stimulation by inflammatory cytokines, while the clinical results of MSC-based therapies for acute GVHD are diverse (2, 3). Those MSCs are not active forms and expected to be active after the administration.

We previously reported that UC-MSCs secreted indoleamine 2,3-dioxygenase-1 (IDO-1), a key soluble factor to inhibit the activated T-cell proliferation, only upon the IFN-γ stimulation or allogenic mixed lymphocyte reaction (MLR), while no IDO-1 expressed without inflammations (4). Several trials have been reported to prime MSCs. Kim et al. reported that IFN-γ primed MSCs significantly reduced the symptoms of GVHD in NOD-SCID mice and prolonged survival rate compared to those with naïve MSCs (5). Wobma et al. also demonstrated that IFN-γ induced IDO-1, programmed cell death-ligand 1 (PD-L1), and human leukocyte antigen G (HLA-G) in MSCs, and it associated with stronger immunosuppression on the activated CD4- and CD8-positive T cells in MLR (6). Combining the hypoxia with IFN-γ priming showed additive effect of IFN-γ alone on T-cell proliferations in MLR, although gene expression of *IDO-1* and *PD-L1* was slightly suppressed by hypoxia.

Cassano et al. reported that MSC exposure to LPS or TLR3 stimulation resulted in more suppressive effects on activated T-cell proliferation (7). Transforming growth factor beta (TGF-β) is constitutively expressed in MSCs, when the lymphocytes are co-cultured with MSCs (8), whereas IFN-γ induces MSCs to have more immunosuppression, possibly by upregulating TGF-β in addition to IDO-1 (4). Superoxide dismutase (SOD) is also secreted by MSCs (9). Elderly MSCs exhibited the downregulation of SOD1 and SOD3, resulting in the elevation of reactive oxygen species (ROS) (10). Jeong et al. reported that ethanol extracts of U. pinnatifida primed BM-MSCs against oxidative injury and upregulated the expression of SOD1 and SOD2. It proved that MSCs expressed factors that could be primed by other irritants (11).

MSCs could, directly and indirectly, change the production of pro-inflammatory cytokines. TNF-α is one of these important cytokines. Beldi et al. reported that TNF/TNFR signaling pathway plays a dual role: interaction between TNF-α and TNF receptor 1 (TNFR1) induces inflammation resulting in cell death, and its interaction with the TNF receptor 2 (TNFR2) induces an anti-inflammatory effect (12). In addition, the TNFR2 axis is an important factor of MSC immunological and regenerative functions. Blocking TNFR2 signaling resulted in diminished expression of MSC surface characteristic markers, reduced MSC colony-forming units, and many other biological functions (13). However, these priming reagents themselves are immune activators. Thus, it will be ideal if MSCs are primed to be activated and ready to suppress the immune system reaction before their *in vivo* administrations by new reagents, not immune activator in itself.

Triptolide (TPL) is a diterpenoid triepoxide purified from the Chinese herb *Tripterygium wilfordii* Hook.f. (TWHF), and its chemical structure is C_20_H_24_O_6_ (14). The root of the TWHF plant, which is known in China as Lei-Gong-Teng, has been used in traditional Chinese medicine for more than 2000 years because of its medicinal properties (15). Its potential therapeutic value was recognized by western medicine only after investigators observed the effectiveness of TWHF in the treatment of leprosy and rheumatoid arthritis (16, 17). TWHF has been shown to possess potent anti-inflammatory and immunosuppressive properties *in vitro* (18). It is also effective in the treatment of a variety of autoimmune diseases and in prevention of allograft rejection and GVHD in both animals and humans (14) and has antitumor effects (19). Although the precise immunological mechanisms of TPL remain unknown. Liu et al. reported that TPL decreased the percentages of CD8+, CD4+/CD8+, Th1/Th2 on pristane treatment of peripheral blood of systemic lupus erythematosus (SLE) BALB/c-nude mice (20). Huang et al. reported that T-cell proliferation was reduced in TPL-treated nonobese diabetic (NOD) mice. TPL treatment increased the apoptosis of T cells in the spleen of recipients and prolongs the survival of islet grafts against autoimmune attack or allograft rejection (21). So, we anticipated that the combination of TPL and MSCs could enhance their immunosuppressive reaction. In previous reports, TPL sometimes revealed severe side effects including cardiac, hematopoietic, and renal dysfunctions in the clinical setting. Therefore, we aimed to use TPL as a priming reagent for UC-MSCs to enter a stronger immunosuppressive status. This study is the first to report on the immunosuppressive effects of TPL-primed UC-MSCs *in vitro.*

## Materials and Methods

### Isolation and Culture of UC-MSCs

This study was carried out in accordance with the Ethics Committee of the Institute of Medical Science, the University of Tokyo, and the NTT Medical Center Hospital and Yamaguchi Hospital, Japan with written informed consent from all subjects. UC-MSCs were provided by cord blood and umbilical cord bank, Research hospital, the Institute of Medical Science, The University of Tokyo (IMSUT CORD), Japan. UC-MSCs were isolated from three individual donors regardless of gender using previously reported methods (22, 23). Briefly, frozen-thawed UC tissues were minced into 2-mm fragments and underwent the improved explant culture procedure. The fragments were aligned at regular intervals in 10-cm culture dishes and covered with Cellamigo (Tsubakimoto Chin Co., Japan) defined as the improved explant method culture procedure as per a previously described method (24). Tissue fragments were cultured in culture medium consisting of α-minimal essential medium (αMEM; Wako Pure Chemical Industries, Ltd., Japan) supplemented with 10% fetal bovine serum (FBS: Cell Culture Bioscience, USA) and antibiotics-antimycotics (Antibiotic-Antimycotic, 100X; Life Technologies, USA) (complete medium) at 37°C with 5% CO_2_. Migrating cells from the UC tissue fragments were harvested using the TrypLE Select (Life Technologies) and were defined as passage 1 (P1) UC-MSCs. The P1 cells were once frozen in Stemcell Banker (Zenoaq, Japan). Frozen-thawed cells (2.5×10^5^) suspended in culture medium were seeded in 10 cm culture dish and further expanded until 80–90% confluency and passaged every 5 days with every 2 days refreshing the medium. P4 cells were used for the following experiments. UC-MSCs were preserved in cryoprotectant, Cellbanker 1 plus (Zenoaq Resource CO., LTD., Japan), and thawed before use.

### Cell Proliferation Assay

To determine the priming condition of TPL, we studied proliferation experiment of UC-MSCs in the presence of various dose of TPL. TPL was dissolved in dimethylsulfoxide (DMSO) at concentration of 1 mM and further diluted with αMEM. DMSO diluted with αMEM at the same concentration was added as control. Cell proliferation was assessed using the Cell Counting Kit-8 (CCK-8) (Dojin Molecular Technologies, Inc., Japan). In brief, cells were suspended in complete medium at a density of 5.0 × 10^4^/ml and seeded (100 μl/well) in a 96-well plate. When cells attached to the bottom of the wells after 3 h, TPL was added at a final concentration of 0.01, 0.1, or 1 μM. Cells were cultured for 24, 48, 72, or 96 h, and the water-soluble tetrazolium salt (WST-8) reagent was added into each well followed by 2 h of incubation in CO_2_ incubator. The optical density of the cells was detected at a wavelength of 450 nm using a microplate reader.

### TPL-Primed UC-MSCs

After determining condition of TPL priming on UC-MSCs, we primed UC-MSCs with TPL as follows. UC-MSCs were cultured for 24 h without TPL, followed by the priming with TPL (Chemscene, NJ) at 0.01 μM for 24 h. Then, the TPL-primed UC-MSCs were washed twice to wash out the TPL containing medium, and resuspended in fresh medium, cultured for 48 h followed by harvesting cells by Trypsin. The harvested cells were used for the following experiments.

### Flow Cytometry Analysis of Surface Markers

Standard flow cytometry (FCM) was used to determine the typical cell surface markers of non-primed UC-MSCs and TPL-primed UC-MSCs. UC-MSCs at P3 were cultured at 0.01 μM of TPL for 24 h and washed and cultured as described above (=P4 UC-MSCs). The cells were stained with the following mouse monoclonal antibodies (mAbs): Phycoerythrin (PE)-conjugated anti-human CD73 (#550257, BD, CA, USA), CD11b (#561001, BD), CD19 (#561741, BD, CA, USA), and HLA-DR (#556644, BD); FITC-conjugated anti-human CD90 (#555595, BD), CD34 (#348053, BD), HLA-ABC [IM1838U, Beckman coulter (BC), CA, USA], and CD44 (#347943, BD); APC-conjugated anti-human CD105 (#562408, BD) and CD45 (#IM2475, BC). FITC (#A07795, BC)-, PE (#A07796, BC)-, and APC (#555751, BD)-IgG were used as isotypic controls. Cell suspensions were stained with the fluorochrome-conjugated antibodies at 4°C for 20 min and washed prior to analysis. Dead cells were identified by staining with 7-Amino-Actinomycin D (7AAD) at room temperature for 15 min. Stained cells were collected with a FACS Canto II (BD) and analyzed with FlowJo Software (Tomy Digital Biology, Co. Ltd., Japan).

### MLR Assay

Cells of the leukemic plasmacytoid dendritic cell line, named PMDC05, which was established by Dr. Narita M and obtained by materials transfer agreement from School of Health Sciences, Faculty of Medicine, Niigata University, were used as the stimulator cells　 (25, 26). MLR assay was described previously (4). Briefly, frozen–thawed human cord blood derived mononuclear cells (CB-MNCs) were used as the responder cells, followed by staining with 5-(and -6)-Carboxyfluorescein diacetate succinimidyl ester (Vybrant CFDA SE Cell Tracer Kit; Invitrogen). On the day of MLR, frozen-thawed PMDC05 were adjusted to 2 × 10^5^ cells/ml, suspended in RPMI 1640 medium supplemented with 10% FBS, irradiated at 50 Gy, not to be proliferated, and kept on ice for use. CFSE-labeled CB-MNC were adjusted to 2 × 10^6^ cells/ml, irradiated PMDC05, and UC-MSCs, or TPL-primed UC-MSCs were mixed at a R:S:MSCs = 10:1:1 ratio in the presence of 1 ng/ml anti-human CD3 antibody. After 4 days of culture, the cells were harvested and stained with PE-Cy7-anti-CD4 (#557852, BD) or APC-anti-CD8 antibodies and 7AAD. The CFSE fluorescence intensities of responder T cells were measured separately for CD4^+^ and CD8^+^ responder cells. The responder cells were also treated with phytohemagglutinin-L (PHA-L; Sigma-Aldrich). In the CFSE histograms, when the cell divides, the fluorescence should be apportioned equally to the daughter cells to make sub-peaks, with roughly half the parental intensity. Parental intensity was the standard line, and the immunosuppressive effects of TPL-primed UC-MSCs and non-primed UC-MSCs were compared by the blockade of the daughter cells peaks. Gating strategies are shown in **Supplementary Figure S1**. Briefly, we gated lymphocyte population, 7AAD negative fraction (alive cells), and CD8-single positive and CD4-single positive fractions and analyzed CFSE intensities.

### RNA Isolation and RT-PCR Analysis

We compared the induction of anti-inflammatory cytokines and molecules in TPL-primed UC-MSCs with that in the non-primed UC-MSCs in response to IFN-γ and/or TNF-α. Briefly, UC-MSCs were cultured for 24 h in the culture medium and exposed to TPL with the addition of 0.01 μM for the next 24 h. The medium was then replaced with fresh culture medium containing 10 ng/ml IFN-γ and/or 15 ng/ml TNF-α. After 48 h of culture, RT-PCR was used to evaluate indoleamine 2,3-dioxygenase-1 (*IDO-1*) (4), superoxide dismutase 1 (*SOD1*), superoxide dismutase 2 (*SOD2*), and *Tgf-β* gene expressions. Total RNA was extracted from non-primed UC-MSCs, TPL-primed UC-MSCs, IFN-γ induced UC-MSCs, and IFN-γ induced TPL-primed UC-MSCs using Nucleospin RNA (Invitrogen Corp, Carlsbad, CA, USA). RT-PCR was performed using PrimeScript™ RT reagent Kit (Takara, Shiga, Japan) according to manufacturer’s instructions. The *IDO-1*, *TGF-β*, *SOD1*, and the control gene glyceraldehyde-3-phosphate dehydrogenase (*Gapdh*) were amplified from the synthesized cDNAs by PCR with the primer pairs. The following human-specific primer sequences were used: *IDO*, forward 5’-GGGACACTTTGCTAAAGGCG-3’, reverse 5’-GTCTGATAGCTGGGGGTTGC-3’; *SOD1*, forward 5’ -CTCTC AGGAGACCATTGCATCA -3’, reverse 5’- CCTGTCTTTGTA CTTTCTTCATTTCCA -3’; *SOD2*, forward 5’- GCAAC TCCCCTTTGGGTTCT -3’, 5’-TATACAAGGTCCATT CCCCCG-3’; *TGF-β*, forward 5’- GCGGC AGCTGTACATTG ACT-3’, reverse 5’- CCACGTAGTACACGATGGG-3’; *Gapdh*, forward 5’-AGCCTCAAGATCATCAGCAATG-3’, reverse 5’-ATGGACTGTGGTCATGAGTCCTT-3’. The amplification conditions included a denaturation step at 95°C for 10 min, followed by 35 cycles of denaturation at 94°C for 30 s, primer annealing at 56°C for 30 s, and primer extension at 72°C for 1 min. The data were analyzed in the Thermo Scientific PikoReal Real Time PCR System (Thermo pikoreal 96).

### West Blotting Analysis

We compared the induction of anti-inflammatory cytokines and molecules in TPL-primed UC-MSCs with that in the non-TPL-primed UC-MSCs in response to IFN-γ and/or TNF-α. UC-MSCs were cultured for 24 h in the culture medium and exposed to TPL with the addition of 0.01 μM for the next 24 h. The medium was then replaced with fresh culture medium containing 10 ng/ml IFN-γ and/or 15 ng/ml TNF-α. After 48 h of culture, the proteins were extracted according to the general procedure and the concentration was measured using BCA protein concentration assay kit (Beyotime biotechnology; China);. Protein samples (50 μg) were loaded on the 10% polyacrylamide gel. After electrophoresis, the proteins were transferred to PVDF membrane pretreated with formaldehyde (transfer condition was 4°C, 90 mA, 1.5 h). Skim milk powder (5%) was sealed for 1 h, and diluted primary antibody IDO was added (Invitrogen; USA), TGF-β (Abcam; USA), SOD1 (Abcam; USA), and SOD2 (Abcam; USA), incubated at room temperature for 2 h, and HRP-labeled secondary antibody against sheep and rabbits (Abcam; USA) and incubated at room temperature for 1 h. The signal was visualized using enhanced chemiluminescence reagent(Biosharp; USA) according to the manufacturer’s protocol. Densitometric analysis was performed using ImageJ software. The expression levels of protein in each sample were normalized to GAPDH (Abcam; USA).

### ELISA Analysis

R, R+S, R+S+MSC, R+S+TPL-primed MSC were harvested on the 4th day of MLR. The supernatant of different group was extracted, and the ELISA kit (Beyotime; China) was used to detect the IL10 cytokines according to the manufacturer’s instructions. The reagents and the test samples were melted at room temperature. The different standards were diluted according to the manufacturer’s instructions, and a blank hole was set up. Standard wells, sample wells to be tested, add 40 μl of sample diluent and 10 μl of sample to be tested to the wells to be tested and mix well; seal the plate with tin foil and incubate at 37°C for 30 min; gently open the sealing membrane and wash the solution. Wash five times and blot the filter paper dry; except for blank wells, add 50 μl of enzyme-labeled reagent to each well; seal the plate with tin foil and incubate at 37°C for 30 min. Wash five times and blot the filter paper; follow the instructions of the kit. Add the color reagent and mix well, keep the color from light at 37°C for 10 min, and add 50 μl reaction stop solution to each well. Within 15 min, zero the blank holes and measure the absorbance (OD value) of each hole at the specified wavelength in sequence.

### PD-L1, PD-L2 Expression Analysis

We evaluated the PD-L1 and PD-L2 expression in TPL-primed UC-MSCs and non-primed UC-MSCs in response to IFN-γ and/or TNF-α. Cells were cultured for 24 h in the culture medium, exposed to 0.01 μM of TPL for 24 h, and the medium was then replaced with fresh medium containing 10 ng/ml IFN-γ and/or 15 ng/ml TNF-α. After 48 h, UC-MSCs and IFN-γ induced UC-MSCs with or without TNF-α were stained with the following mouse monoclonal antibodies (mAbs): PE-conjugated anti-human PD-L1 (#557924, BD) and APC-conjugated anti-human PD-L2 (#APC-PD-L2, BD); PE- and APC-IgG (Beckman) were used as isotypic controls. Dead cells were identified by staining with 7-Amino-Actinomycin D (7AAD). The stained cells were collected with a FACS Canto II (BD) and analyzed with the FlowJo Software (Tomy Digital Biology, Co. Ltd., Japan).

### Statistical Analysis

The Shapiro-Wilk test was used to test the normality of the data, and the data were analyzed using the one-way ANOVA followed by the Tukey’s multiple comparisons test for normally distributed data and the Dunn’s multiple comparisons test for not-normally distributed data. The GraphPad Prism 8 software was used for all statistical analyses. Data have been presented as mean ± SD, and p < 0.05 was considered statistically significant.

## Results

### Effect of TPL on UC-MSC Proliferation

**Figure 1:**
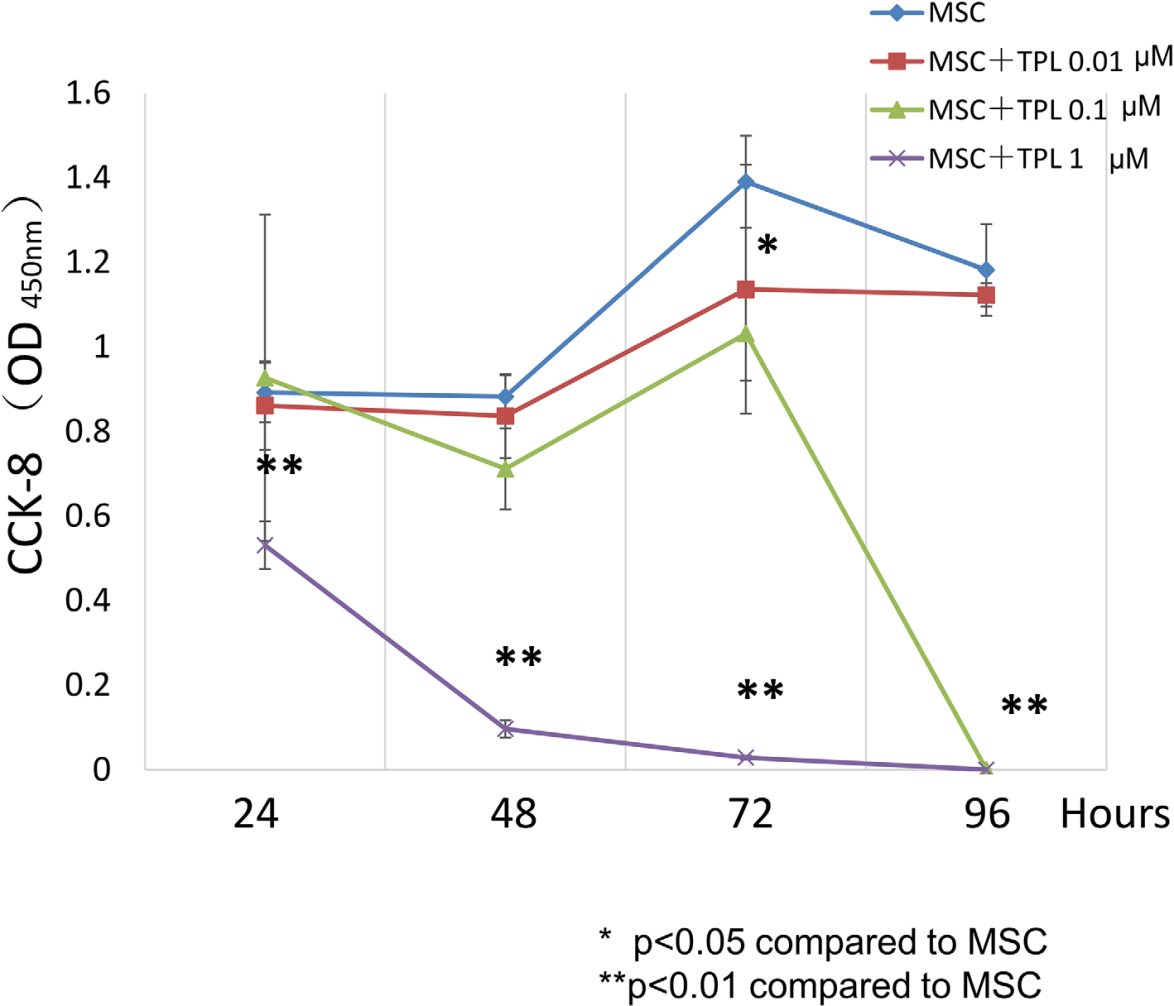
Effect of TPL on the UC-MSC proliferation. CCK8 cell viability assay performed 24, 48, 72, and 96 h after treatment with 1, 0.1, and 0.01 μM TPL; non-primed UC-MSCs were used as the control group. Quantitative data have been presented as the mean value of three different samples ± SD with **p < 0.01, compared to the UC-MSC control group. MSC, UC-MSCs; TPL, Triptolide.

To decide the appropriate dose and duration for TPL priming, UC-MSCs were treated with 0, 0.01, 0.1, and 1 μM of TPL and measured with the CCK-8 viability assay at 24, 48, 72, and 96 h after the TPL treatment (**Figure 1**). Cell proliferation was suppressed significantly by the addition of TPL at a concentration of 0.1 and 1 μM at 96 h. Only 0.01 μM of TPL-primed UC-MSCs showed relatively lower proliferation without significant differences up to 48 h of incubation. Therefore, we decided to perform all subsequent experiments with UC-MSCs primed with 0.01 μM TPL for 24 h (referred as TPL-primed UC-MSCs).

### Expression of Surface Markers of TPL-Primed UC-MSCs

**Figure 2:**
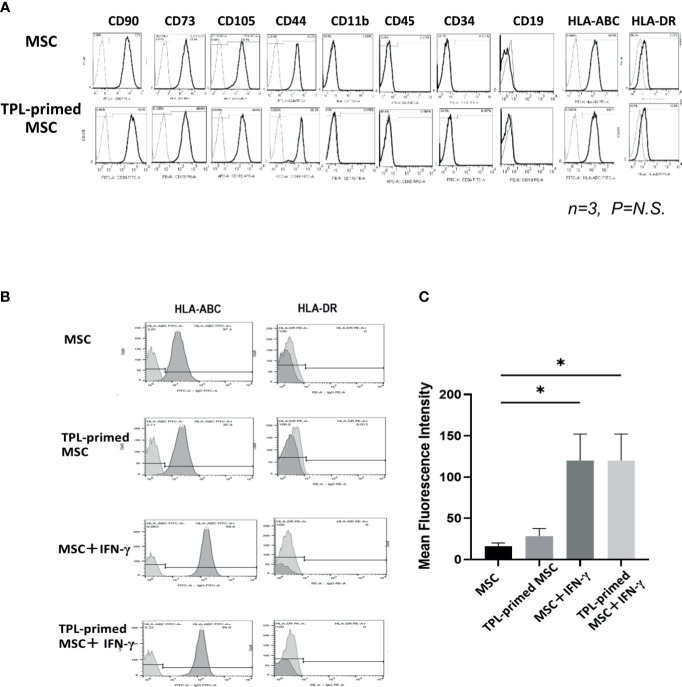
The surface markers of TPL-primed UC-MSCs. **(A)** TPL-primed UC-MSCs express the same surface markers as the UC-MSCs which are positive for CD90, CD73, CD105, CD44, and HLA-ABC, and negative for CD34, CD45, CD11b, and HLA-DR. Mean fluorescent intensity (MFI) of surface marker on MSCs and TPL-primed MSC is shown. There are no significant differences of MFI between TPL-primed UC-MSC (+) and non-primed UC-MSC (-). **(B)** Surface markers of HLA-ABC and HLA-DR in TPL-primed UC-MSCs and UC-MSCs with or without IFN-γ. **(C)** Quantitative analysis of mean fluorescent intensity HLA-ABC in TPL-primed UC-MSCs and UC-MSCs with or without IFN-γ. Quantitative data have been presented as the mean value of three different samples ± SD with *p < 0.05.

We compared the surface makers between non-primed UC-MSCs and TPL-primed UC-MSCs by flow cytometry. Similar to the UC-MSCs, TPL-primed UC-MSCs were positive (>95%) for CD105, CD73, CD44, CD90, and HLA-ABC, and negative (<5%) for CD45, CD34, CD11b, CD19, and HLA-DR (n = 3) (**Figure 2A**). No significant influences on the mean fluorescence intensity (MFI) of these surface molecules by TPL-priming were observed (**Supplementary Table S1**). We also investigated the HLA-ABC expression in TPL-primed UC-MSCs treated with IFN-γ. IFN-γ increased the fluorescent strength of HLA-ABC expression significantly in non-primed UC-MSCs and TPL-primed UC-MSCs, while TPL did not affect this expression (**Figures 2B, C**). These data were also supported by gene expression analysis by microarray (**Supplementary Table S2**).

### The Immunosuppressive Effect of TPL-Primed UC-MSCs

**Figure 3:**
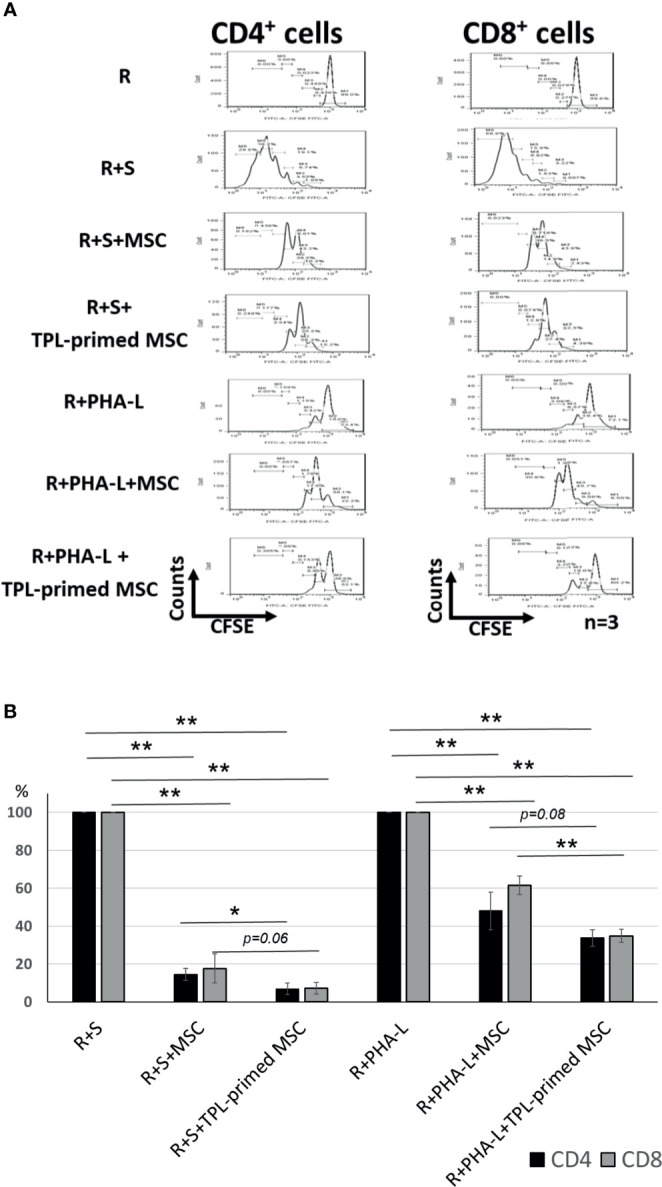
The immunosuppression effect of TPL-primed UC-MSCs. **(A)** TPL-primed UC-MSCs show stronger anti-proliferative effect for activated CD4+ and CD8+ T cells in allogeneic MLR compared with the non-primed UC-MSCs. TPL-primed UC-MSCs exhibit stronger anti-proliferative effect for activated CD4+ and CD8+ T cells on PHA-L stimulated responder cells. **(B)** Comparison of the (%) inhibition of TPL-primed UC-MSCs and UC-MSCs in allogeneic MLR. There are significant differences of inhibitory effect by UC-MSC with or without TPL priming. Quantitative data have been presented as the mean value of three different samples ± SD with *p < 0.05, **p < 0.01.

The immunosuppressive effect of TPL-primed UC-MSCs was compared with that of non-primed UC-MSCs using MLR assays in which PMDC05 and PHA-L were used as stimulators. TPL-primed UC-MSCs exerted significantly stronger anti-proliferative effect on activated CD4+ and CD8+ T cells than the UC-MSCs alone (n = 3, **Figure 3A**). The proliferations of activated CD4+ and CD8+ T cells stimulated by the allogeneic PMDC05 in the co-culture with non-primed UC-MSCs were suppressed to 14.49% ± 3.11% and 17.64% ± 7.69%, respectively, and in the R+S+TPL-primed MSCs were suppressed to 6.88% ± 3.01% and 7.26% ± 3.03%, respectively. The proliferations of activated CD4+ and CD8+ T cells stimulated by PHA-L in the co-culture with non-primed UC-MSCs were suppressed to 48.07% ± 9.88% and 61.52% ± 4.95%, respectively, and in R+S+TPL-primed MSCs were suppressed to 33.72% ± 4.27% and 34.86% ± 3.41%, respectively (n = 3, **Figure 3B**).

### Gene Expression in TPL-Primed UC-MSCs in the Presence of IFN-γ/TNF-α

**Figure 4:**
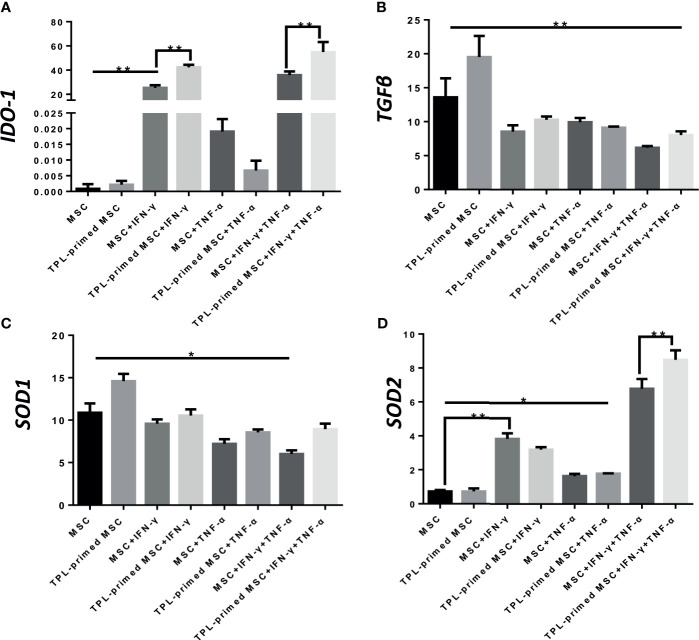
Gene expression of suppressive molecules in TPL-primed or non-primed UC-MSCs in the presence of IFN-γ and/or TNF-α. The expression levels of IDO-1, TGF-β, SOD1, and SOD2 **(A-D)** in TPL-primed UC-MSCs with IFN-γ/TNF-α were examined by RT-PCR. Quantitative data have been presented as the mean value of three different samples ± SD with *p < 0.05, **p < 0.01.

Next, we evaluated the *IDO-1*, *SOD1*, *SOD2*, and *TGF-β* gene expressions in TPL-primed UC-MSCs with IFN-γ/TNF-α in comparison with that in the non-primed UC-MSCs (**Figures 4A–D**). We found that TPL-primed UC-MSCs promoted the expression of *IDO-1* when stimulated by IFN-γ regardless of TNF-α, while TPL alone did not induce the *IDO-1* expression. *SOD1* was expressed constitutively in non-primed UC-MSCs, and TPL had a tendency to further induce its expression, but that was not statistically significant. *SOD2* in non-primed UC-MSCs was induced in the presence of IFN-γ, and IFN-γ and TNF-α synergistically induced *SOD2* significantly. Non-primed UC-MSCs expressed *TGF-β* constitutively, and its expression was further induced by TPL priming, whereas the addition of IFN-γ and/or TNF-α suppressed this induction.

### Protein Expression Levels in TPL-Primed UC-MSCs in the Presence of IFN-γ/TNF-α

**Figure 5:**
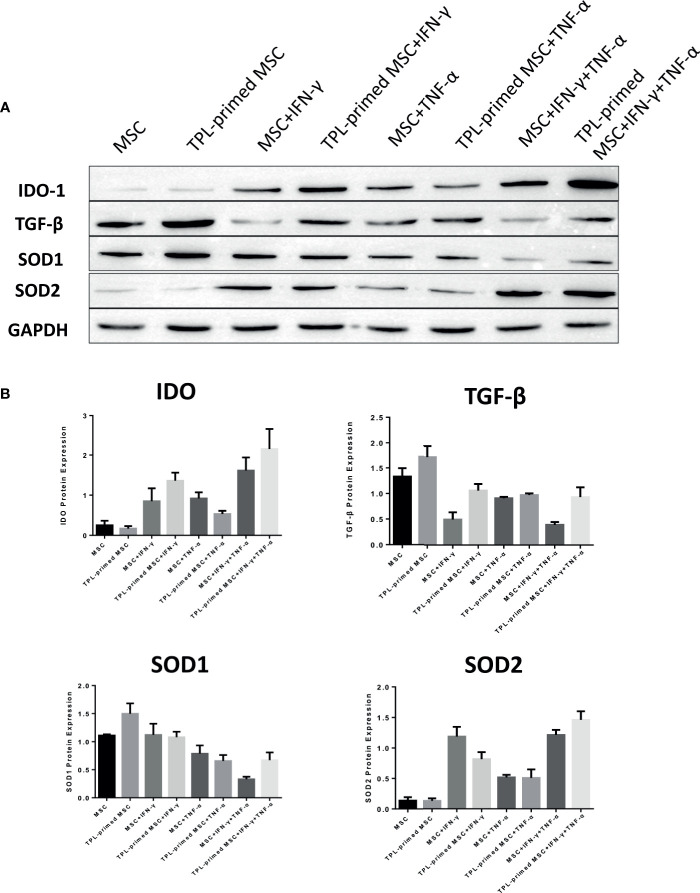
Protein expression in TPL-primed or non-primed UC-MSCs in the presence of IFN-γ and/or TNF-α. **(A)** The protein expression of IDO-1, TGF-β, SOD1, and SOD2 in TPL-primed UC-MSCs and non-primed UC-MSCs in response to IFN-γ and TNF-α by western blotting. The representative data of three independent experiments are shown. **(B)** Quantitative data corresponding to GAPDH expression are presented as the mean value of three different samples ± SD with the *P* value tables (**Supplementary Figure S2**).

We evaluated the protein expression levels of IDO-1, SOD1, SOD2, and TGF-β in TPL-primed UC-MSCs with IFN-γ/TNF-α in comparison with those in the non-primed UC-MSCs (**Figures 5A, B**). Significant values are shown in **Supplementary Figure S2**. TPL priming alone did not induce the IDO-1 at all, while IFN-γ is the strong inducer of IDO-1. We observed the tendency of IDO-1 protein expression induced by TPL priming in the presence of IFN-γ, although there were no significant differences. TGF-β in TPL-primed UC-MSCs expressed significantly more than that in non-primed UC-MSCs. On the other hand, IFN-γ suppressed the TGF-β protein, while TPL priming ameliorated the TGF-β in the presence of IFN-γ. CD4+FOXP3+ regulatory T (Treg) cells were induced in the peripheral blood MNCs (PBMCs), when PBMCs were co-cultured with UC-MSCs for 10 days in RPMI medium supplemented with 10% FBS (**Supplementary Figures S3A, B**). SOD1 was also expressed constitutively in non-primed UC-MSCs, and TPL priming induced more expression of SOD1, significantly. But IFN-γ and TNF-α did not induce SOD1, instead diminished the expression. On the other hand, SOD2 levels were low in UC-MSCs and TPL-primed MSCs. SOD2 was induced significantly by IFN-γ, but no additional effect of SOD2 induction by TPL priming. These protein expression data showed the same tendency corresponding to the results of gene expressions in **Figure 4**.

### IL10 Expression in TPL-Primed UC-MSCs

We checked IL-10 in the MLR experiment by ELISA method. It was observed that as compared with the R+S+MSC group, TPL acted in conjunction with the R+S+MSC group to significantly upregulate IL10 levels (**Supplementary Figure S4**).

### PD-L1, PD-L2 Expression in TPL-Primed UC-MSCs in the Presence of IFN-γ/TNF-α

**Figure 6:**
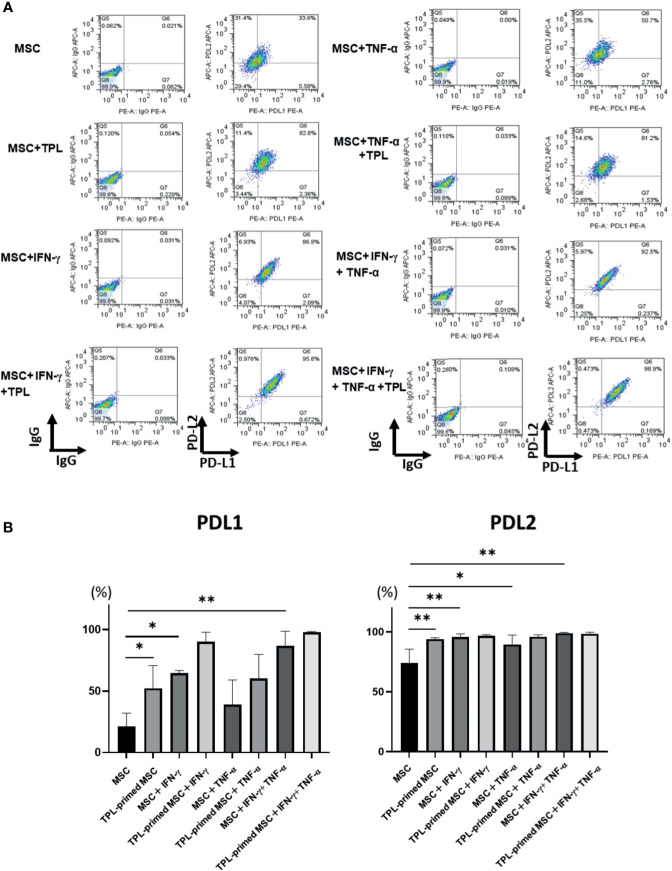
PD-L1 and PD-L2 expression in TPL-primed or non-primed UC-MSCs in the presence of IFN-γ and/or TNF-α. PD-L1 and PD-L2 expression in TPL-primed UC-MSCs and non-primed UC-MSCs in response to IFN-γ and TNF-α. **(A)** FACS analysis of PD-L1 and PD-L2 expression. The representative data of three independent experiments are shown. **(B)** Quantitative analysis. Quantitative data have been presented as the mean value of three different samples ± SD with *p < 0.05, **p < 0.01. MSC, UC-MSCs.

Finally, we investigated the protein expression of PD-L1 and PD-L2 in TPL-primed UC-MSCs compared to that in non-primed UC-MSCs in response to IFN-γ and TNF-α by flow cytometry. PD-L1 and PD-L2 were induced by both IFN-γ and TNF-α. Interestingly, we found that TPL alone could also enhance significantly the expression of PD-L1 and PD-L2, similar to IFN-γ and TNF-α (**Figures 6A, B**).

## Discussion

The immunosuppressive potency of MSCs depends on the inflammatory factors, including those responsible for allogeneic reactions in acute GVHD and autologous diseases. Inflammatory factors such as IFN-γ can induce MSCs to exert their immunosuppressive potency.

TPL itself possesses immunosuppressive properties and has anti-tumor effects (27). TPL was reported to prevent GVHD *in vivo* (28) and to induce allogeneic tolerance in bone marrow transplantation setting (29). Moreover, TPL can enhance apoptosis in tumor cells by blocking p21-mediated growth arrest (19). Although TPL was a likely candidate for clinical use, severe adverse events including myocardial damage, renal failure, and hypovolemic shock secondary to severe intestinal tract disturbances (27) resulted in the limitation of TPL on clinical applications. It has been reported the genotoxic effects of TPL. TPL shows cytotoxicity through inducing DNA damage, increasing sensitivity to DNA-damage based chemotherapy (30)or radiotherapy (31) in different types of cells. Cai et al. demonstrated that treating cells with TPL induced genomic instability when they used human fibroblast cell line, HCA2-hTERT cells. TPL impairs DNA repair and destabilizes genomes more by directly targeting the critical NHEJ factor DNA-PKcs compared with those in control cells at 24 h post ionizing radiation (32). Although the administered MSCs were considered to be eliminated in the patients without engraft, the caution must be paid to clinical applications using TPL (23). In the clinical setting, it may better to check the chromosomal analysis, and other genomic instability tests for the final products of TPL-primed UC-MSCs.

As TPL has both immunosuppressive activity and antiproliferative effect, we first studied the influence of TPL on the proliferation of UC-MSCs. TPL suppressed UC-MSC proliferation even at a low dose of 0.01 μM, but it was tolerable for the first 24–48 h of exposure. Therefore, we decided to prime UC-MSCs with TPL at a dose of 0.01 μM for 24 h. TPL priming did not affect the expression of the defined surface markers for MSCs. The mean intensity of HLA-ABC expression by flowcytometry was upregulated by the addition of IFN-γ, but not by TPL priming. It was reported that the donor cell-mediated anti-leukemic effect on HSCT, donor cell plays a part in antitumor responses by cytotoxic T lymphocytes (CTL) (33). Previously, Huang et al. demonstrated that increase of HLA-class I intensity is related to the antigen presentation ability, resulting in CTL responses (34). It might be better not to enhance the HLA antigen in the GVHD setting, although co-stimulatory factors such as CD80 and CD86 are negative in MSCs. And the most important point is that the negativity of HLA-DR was not influenced by the addition of neither IFN-γ nor TPL priming in UC-MSCs.

Interestingly, we found that TPL-primed UC-MSCs showed stronger antiproliferative effect for activated CD4+ and CD8+ T cells in allogeneic MLR and PHA-L than the non-primed UC-MSCs. To exclude the possibility that TPL directly suppressed the activated T cells by allogeneic dendritic cells (DC) or PHA-L, we thoroughly washed out the TPL after priming and confirmed that the residual TPL alone after the priming and washing had no influence on the additional inhibitory effect observed with the MLR assay (data not shown). Several mechanisms may account for the additional inhibitory effect of TPL-primed UC-MSCs. First, this may be due to the additional induction of IDO-1 secretion. IDO-1 is one of the critical functional factors that inhibit the proliferation of activated T cells and its expression is induced by IFN-γ and other molecules. Its inhibitory effect on the UC-MSCs was partially reversed by the addition of 1-methyl-DL-tryptophan (1-MT), an inhibitor of IDO-1, in a dose-dependent manner (4). TPL priming exaggerated *IDO-1* gene expression in UC-MSCs in response to IFN-γ compared with that in non-primed UC-MSCs, although TPL alone did not induce *IDO-1*. TNF-α did not induce the expression of *IDO-1*; however, it had a synergistic effect with IFN-γ on *IDO-1* induction, which was further enhanced by TPL.

SOD1 and SOD2 are also critical enzymes, that decompose superoxide radicals generated at the site of inflammation (35). SOD1, located in the cytoplasm (36), was expressed constitutively in non-primed UC-MSCs, and TPL induced SOD1 significantly in protein level. SOD2 is expressed in the mitochondria (37) and was induced in non-TPL primed UC-MSCs in the presence of IFN-γ. Importantly, IFN-γ and TNF-α synergistically induced SOD2 in the UC-MSCs, and this was significantly augmented by TPL priming. Non-TPL-primed UC-MSCs expressed and secreted TGF-β constitutively, which is considered to induce the regulatory T (Treg) cells in the presence of IL-2 (38). In this study, we found that TPL induced significantly the expression of TGF-β and expected more Treg induction. However, we did not observe any additional expansion of the Treg cells in TPL-primed UC-MSCs than those in the non-primed UC-MSCs co-cultured with PBMCs. The reason for TPL-primed UC-MSCs not inducing the regulatory T cells despite their higher amount of TGF-β may be the inhibition of IL-2 transcription by TPL (39). IL-10 is an important cytokine that secreted from MSCs, and it is considered an anti-inflammatory molecule. IL-10 can induce macrophage from M1 to M2, which lead to the suppression of pro-inflammatory cytokines (40). We found IL-10 level was upregulated in the MLR cocultured with UC-MSC, and more in TPL-primed UC-MSC, although we need to study the influence of IL-10 secreted from TPL-primed UC-MSC on the polarization of macrophage and MLR suppression.

MSCs have been reported to secrete PD-L1 and PD-L2 which suppress the activation of T cells and downregulate the IL-2 secretion resulting in cell death (41). Both PD-L1 and PD-L2 are reported to be upregulated in response to proinflammatory cytokines such as IFN-γ (42). Witte et al. demonstrated that IFN-γ, TGF-β, and retinoic acid upregulated the expression of immunomodulatory factor PD-L1 and IDO-1 activity (41, 42). UC-MSCs inhibited the differentiation of PBMCs into dendritic cells and induced Tregs through PD-1/PD-L1 pathway (43). Davies et al. have reported that MSCs express and secrete PD-L1 and PD-L2 and that this is regulated by exposure to IFN-γ and TNFα (41). In our experiment, PD-L1 was expressed only at low levels and was induced by TPL priming in UC-MSCs. There were several reports that TPL suppressed the PD-L1 expression in cancer cells by inhibiting IFN-γ secretion (44–46). They suggested the relation between PD-L1 and IFN-γ secretion. However, as far as we know, PDL-1 induction by TPL alone in MSCs has not been reported yet and this is the first report, although the mechanism remained to be elucidated.

In this study, we started the experiments, based on the fact that the proliferation of CD4+ and CD8+ T cells in allogeneic MLR was inhibited more by TPL-primed UC-MSCs effect rather than those by non-primed UC-MSCs. So we have focused T cells and related molecules in the study. But as well known, not only CD4+ and CD8+ T cells, but also M1/M2 monocytes and NK cells play an important role in the pathogenesis of acute GVHD, while the immune system dysregulation is different and complex by the type of acute GVHD (47). Further studies are necessary to elucidate the mechanisms of the influence of TPL-derived UC-MSCs on immune system network.

The results presented in this study suggest that TPL-primed UC-MSCs exert increased immunosuppressive potency on the proliferation of activated T cells, by upregulating IDO-1 in the presence of IFN-γ and by inducing the PD-L1 and TGF-β expressions. Thus, TPL-primed UC-MSCs may have become ready to exert their function. This is the first report of TPL affecting the function of MSCs. The activation or pre-activation mechanism of TPL on MSCs seems to be different from that by IFN-γ.

Different from proinflammatory molecules, IFN-γ, TPL itself is an anti-inflammatory molecule, which could inhibit the infiltration of lymphocytes and the expression of inflammatory factors through JAK/STAT pathway and NF-κB pathway (48). Even if the residual reagent is contaminated in the final product, TPL might be safer than IFN-γ. Thus, TPL could be alternative priming reagent to IFN-γ. The exact molecular details of the TPL-induced activation of UC-MSCs needs to be investigated further.

Conclusively, TPL- primed UC-MSCs may provide the means for a novel immunosuppressive cell therapy.

## Data Availability Statement

The raw data supporting the conclusions of this article will be made available by the authors, without undue reservation.

## Ethics Statement

This study was carried out in accordance with the Ethics Committee of the Institute of Medical Science, the University of Tokyo, and the NTT Medical Center Hospital and Yamaguchi Hospital, Japan with written informed consent from all subjects.

## Author Contributions

All authors listed have made a substantial, direct, and intellectual contribution to the work and approved it for publication.

## Funding

This work was supported by the Sasagawa scholarship grant, National Natural Science Foundation of China (NSFC-81760028), Yunnan Provincial Basic Research (Kunming Medical Joint Project-2018FE001-129), and Training objects of medical subject leaders in Yunnan Province(D-2018017). This study was also supported by the IMSUT International Joint Research Project grant with The Affiliated Hospital of Kunming University of Science and Technology (Project Number: New 2019-1027 and New-2020-K1006).

## Conflict of Interest

The authors declare that the research was conducted in the absence of any commercial or financial relationships that could be construed as a potential conflict of interest.

## Publisher’s Note

All claims expressed in this article are solely those of the authors and do not necessarily represent those of their affiliated organizations, or those of the publisher, the editors and the reviewers. Any product that may be evaluated in this article, or claim that may be made by its manufacturer, is not guaranteed or endorsed by the publisher.

## Supplementary Material

Supplementary Figure 1 The gating strategies for analysis of CFSE-stained lymphocytes in MLR. Gating strategies of allogeneic MLR against activated CD4+ and CD8+ T cells using UC-MSCs. After lymphocyte population in side scatter (SSC) and forward scatter (FSC), 7-Amino-Actinomycin D (7AAD) negative fraction (alive cells) are gated, and CD8-single positive and CD4-single positive fractions are analyzed with 5-(and -6)-Carboxyfluorescein diacetate succinimidyl ester (CFSE) intensities.

Supplementary Figure 2 Protein expression levels in TPL-primed UC-MSCs in the presence of IFN-γ/TNF-α with significant value tables.

Supplementary Figure 3 Regulatory T cells in the mononuclear cells co-cultured with UC-MSC and TPL-primed UC-MSC by flow cytometry analysis. **(A)** Gating strategies of FACS analysis of regulatory T cells in the mononuclear cells co-cultured with UC-MSC and TPL-primed UC-MSCs. **(B)** Percentage of regulatory T cells in the mononuclear cells co-cultured with UC-MSC and TPL-primed UC-MSCs. Quantitative data have been presented as the mean value of three different samples ± SD.

Supplementary Figure 4 IL-10 levels in MLR by ELISA. The IL-10 levels in the supernatant of mixed lymphocyte reaction (MLR) were evaluated by ELISA. IL-10 levels were upregulated in R+S+MSC group, more in R+S+TPL-primed UC-MSC (MSC) compared with those in R+S alone. In the one-way analysis of variance between the model group and the control group (R+S), * indicates p < 0.05 or ** indicates p < 0.01, ***<0.001.

Supplementary Table 1 Mean fluorescent intensity of surface marker on UC-MSCs and TPL-primed UC-MSCs.

Supplementary Table 2 The gene expressions of HLA-A, HLA-B, and HLA-C in UC-MSCs by microarray analysis. Microarray analysis of the gene expressions of HLA-A, HLA-B, and HLA-C in TPL-primed UC-MSCs, IFNγ+ UC-MSCs, and IFN-γ+ TPL-primed UC-MSCs *versus* UC-MSCs as control. The values are shown by log2 ratio.
