# Supplementary figures and images for: The Immunomodulatory Effect of Triptolide on Mesenchymal Stromal Cells
Source: Front Immunol. 2021 Aug 16;12:686356. doi: 10.3389/fimmu.2021.686356 (PMC8415460; doi:10.3389/fimmu.2021.686356)

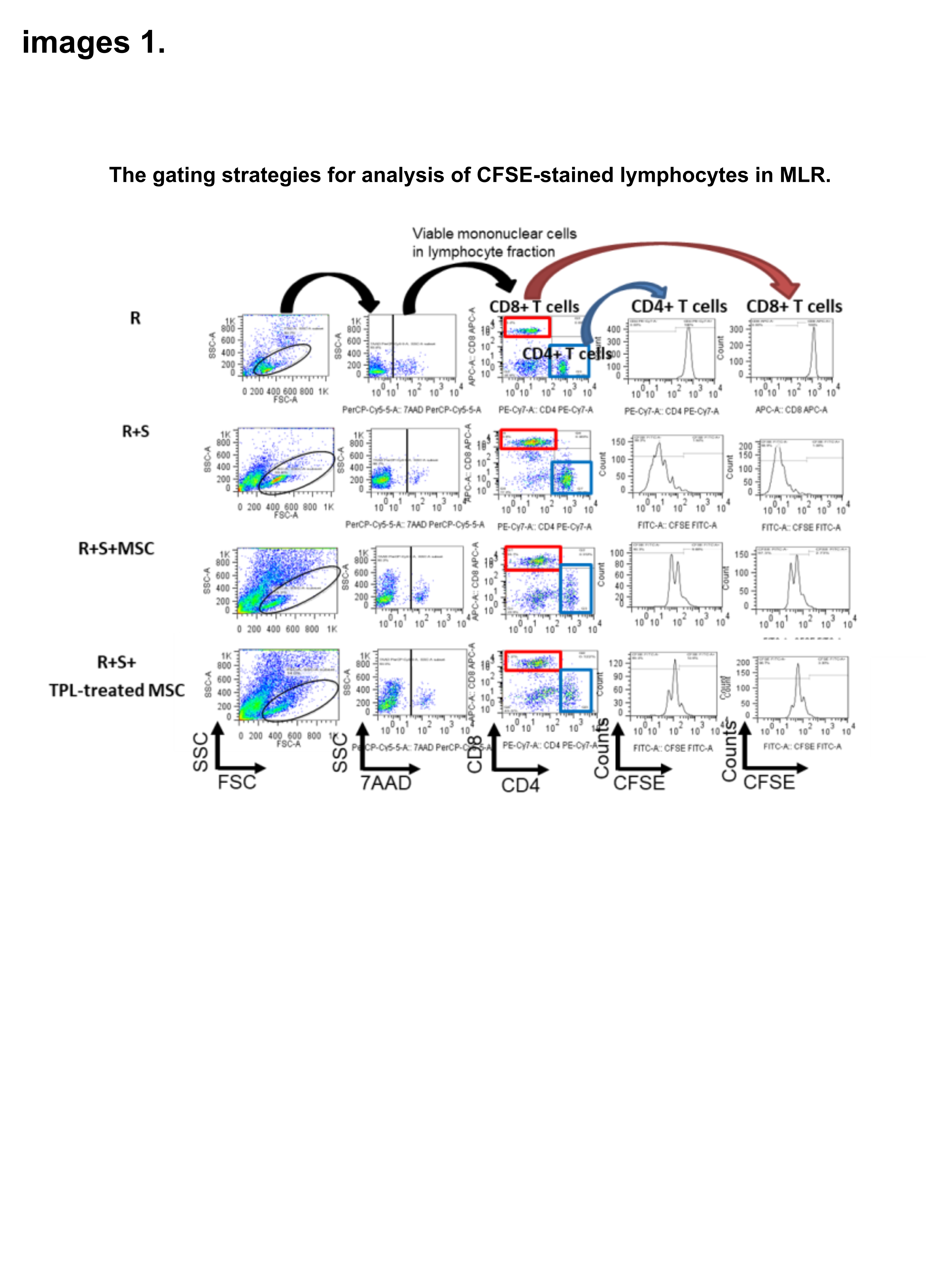

Supplement: Supplementary Figure 1 — The gating strategies for analysis of CFSE-stained lymphocytes in MLR. Gating strategies of allogeneic MLR against activated CD4+ and CD8+ T cells using UC-MSCs. After lymphocyte population in side scatter (SSC) and forward scatter (FSC), 7-Amino-Actinomycin D (7AAD) negative fraction (alive cells) are gated, and CD8-single positive and CD4-single positive fractions are analyzed with 5-(and -6)-Carboxyfluorescein diacetate succinimidyl ester (CFSE) intensities. [file Image_1.jpg]

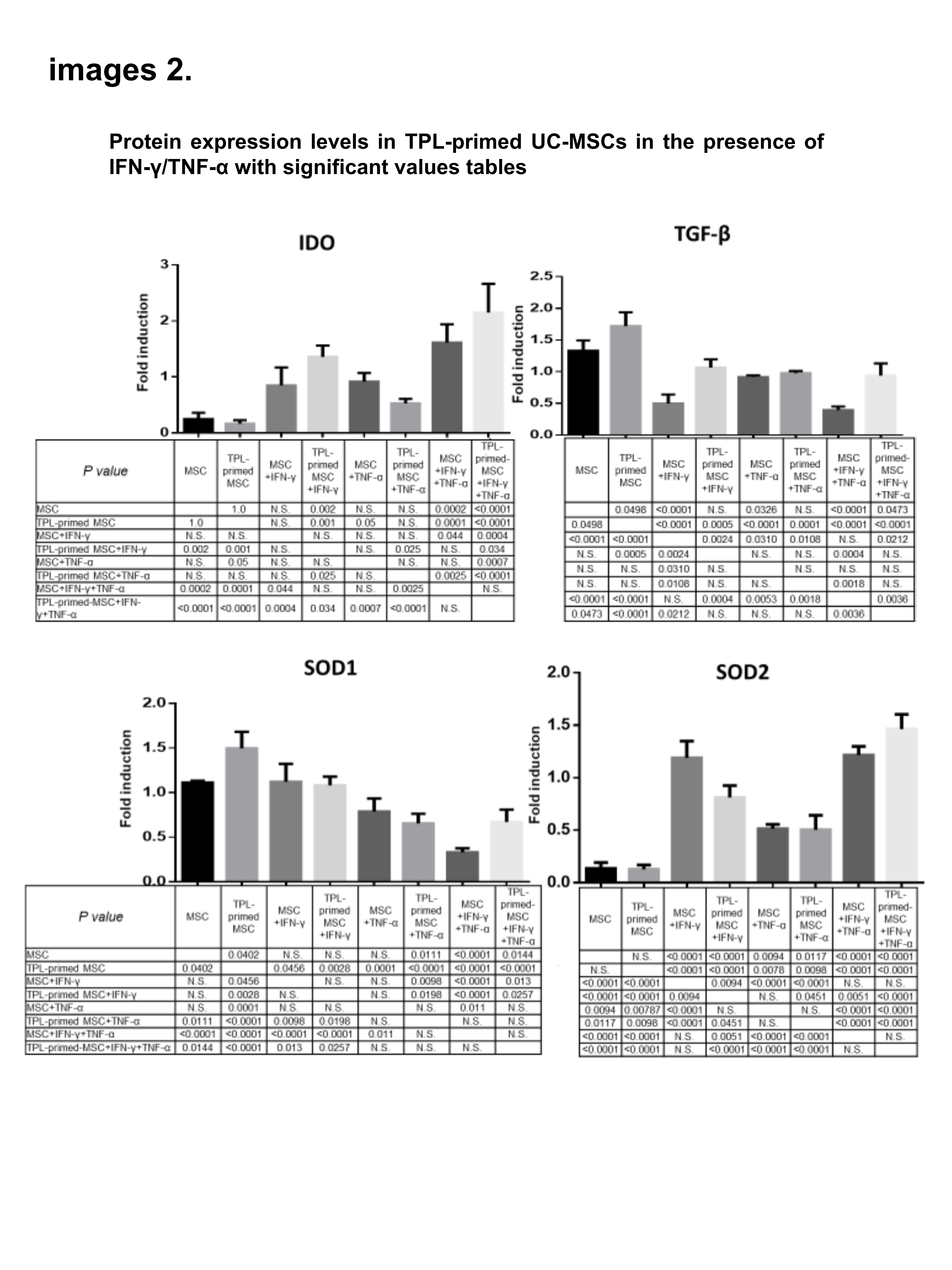

Supplement: Supplementary Figure 2 — Protein expression levels in TPL-primed UC-MSCs in the presence of IFN-γ/TNF-α with significant value tables. [file Image_2.jpg]

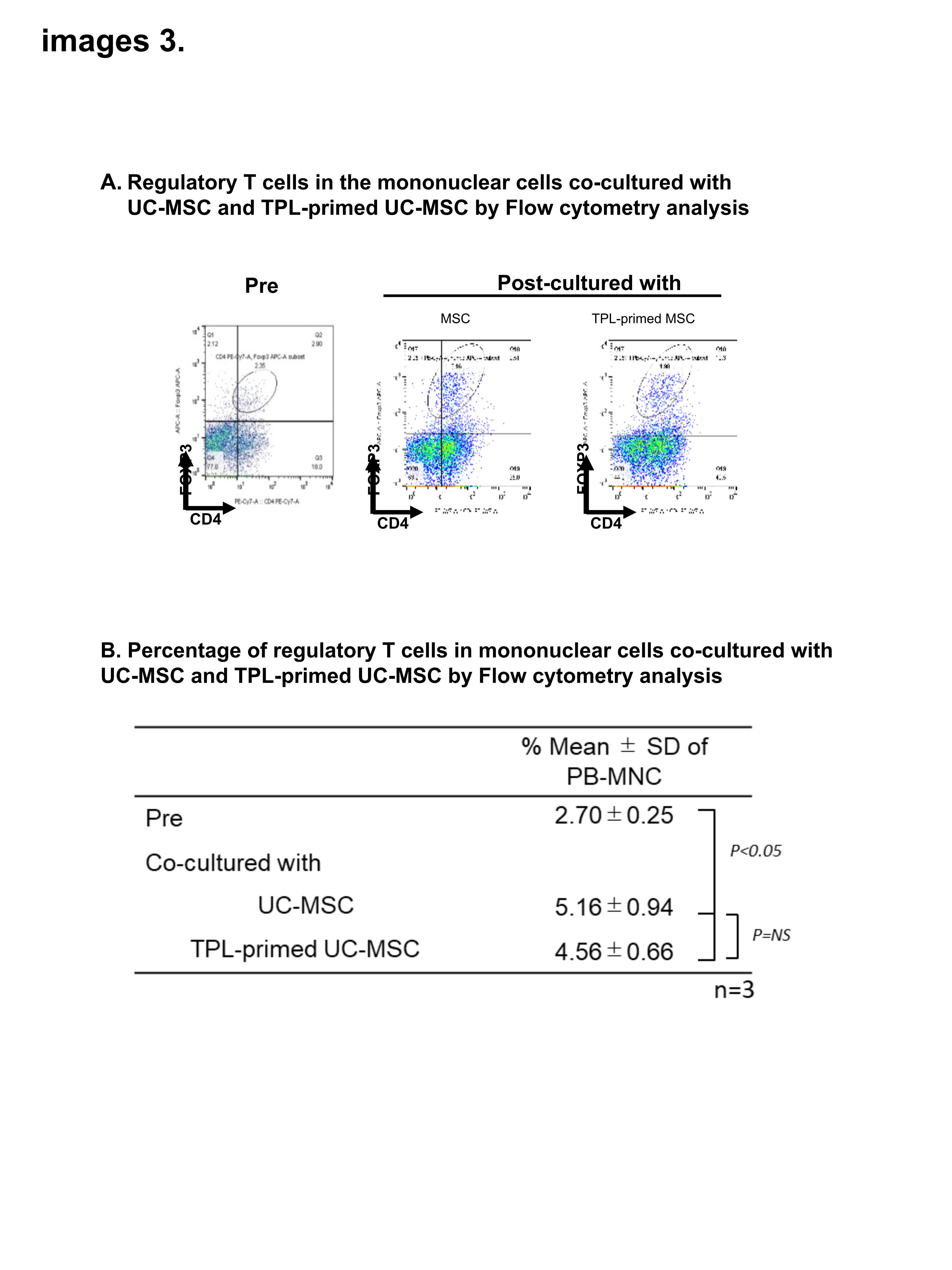

Supplement: Supplementary Figure 3 — Regulatory T cells in the mononuclear cells co-cultured with UC-MSC and TPL-primed UC-MSC by flow cytometry analysis. (A) Gating strategies of FACS analysis of regulatory T cells in the mononuclear cells co-cultured with UC-MSC and TPL-primed UC-MSCs. (B) Percentage of regulatory T cells in the mononuclear cells co-cultured with UC-MSC and TPL-primed UC-MSCs. Quantitative data have been presented as the mean value of three different samples ± SD. [file Image_3.jpg]

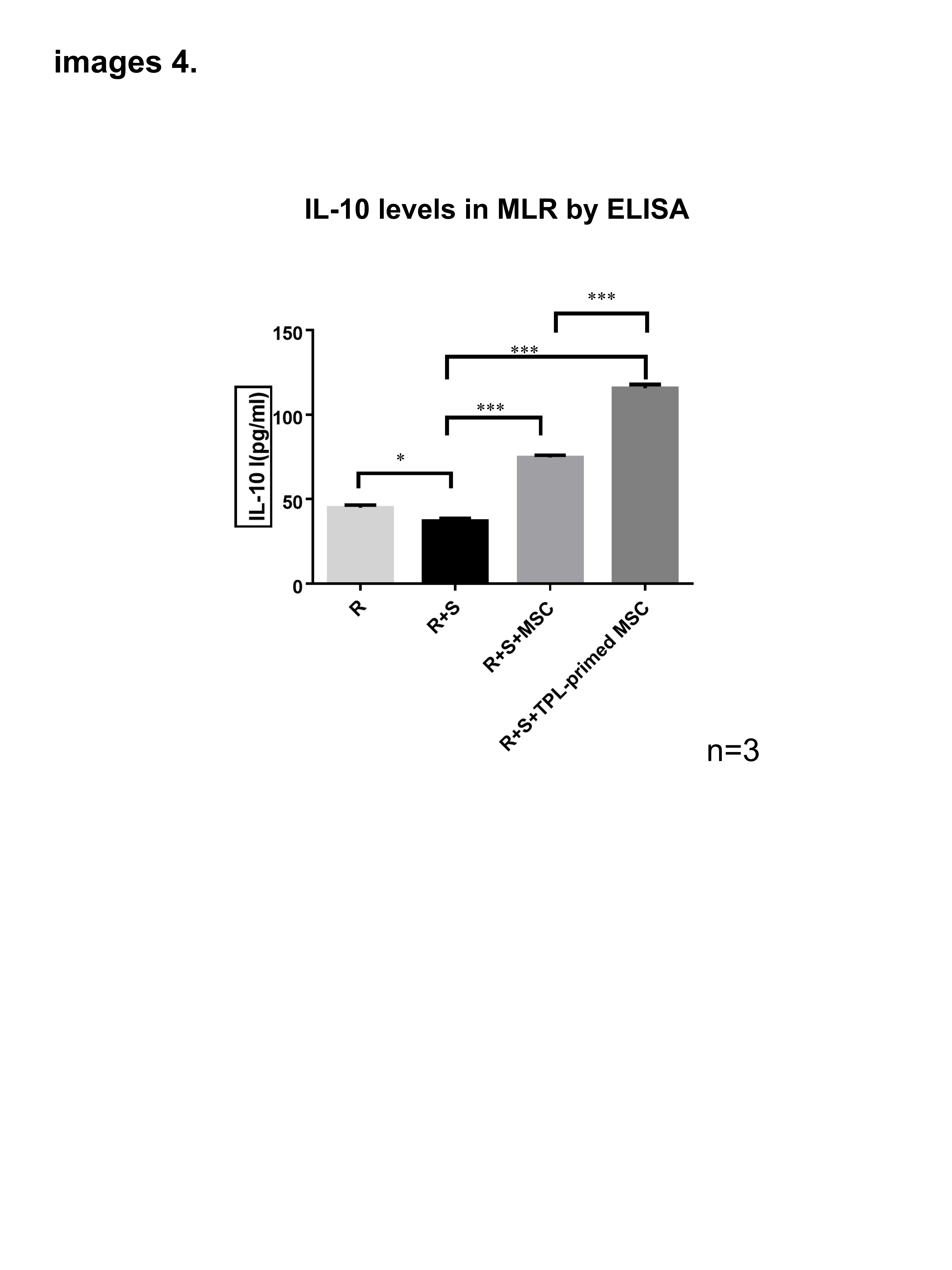

Supplement: Supplementary Figure 4 — IL-10 levels in MLR by ELISA. The IL-10 levels in the supernatant of mixed lymphocyte reaction (MLR) were evaluated by ELISA. IL-10 levels were upregulated in R+S+MSC group, more in R+S+TPL-primed UC-MSC (MSC) compared with those in R+S alone. In the one-way analysis of variance between the model group and the control group (R+S), * indicates p < 0.05 or ** indicates p < 0.01, ***<0.001. [file Image_4.jpg]
