# Supplementary material for: The Immunomodulatory Effect of Triptolide on Mesenchymal Stromal Cells
Source: Front Immunol. 2021 Aug 16;12:686356. doi: 10.3389/fimmu.2021.686356 (PMC8415460; doi:10.3389/fimmu.2021.686356)
Supplement: Supplementary Table 1 — Mean fluorescent intensity of surface marker on UC-MSCs and TPL-primed UC-MSCs. [file Table_1.pdf]

Table1.

Mean fluorescent intensity of surface marker on UC-MSCs and TPL-primed UC-MSCs

	CD73		CD105		CD90		CD44		HLA-ABC		CD45		CD34		CD11b		CD19		HLA-DR	
TPL-primed	-	+	-	+	-	+	-	+	-	+	-	+	-	+	-	+	-	+	-	+
M.F.I. Mean ±SD	169.3 ±10.5	166.0 ±7.2	226.0 ±7.1	214.5 ±14.9	471.50 ±45.9	483.0 ±28.3	289.0 ±14.5	266.3 ±30.6	181.3 ±16.8	184.0 ±14.5	5.2 ±1.1	5.2 ±0.9	6.3 ±1.0	7.1 ±2.4	6.5 ±0.4	6.9 ±0.6	13.6 ±9.5	13.5 ±8.6	10.5 ±5.6	11.1 ±5.8

Mean fluorescent intensity; M.F.I., n=3, *p=not significant*
