# Supplementary material for: The Immunomodulatory Effect of Triptolide on Mesenchymal Stromal Cells
Source: Front Immunol. 2021 Aug 16;12:686356. doi: 10.3389/fimmu.2021.686356 (PMC8415460; doi:10.3389/fimmu.2021.686356)
Supplement: Supplementary Table 2 — The gene expressions of HLA-A, HLA-B, and HLA-C in UC-MSCs by microarray analysis. Microarray analysis of the gene expressions of HLA-A, HLA-B, and HLA-C in TPL-primed UC-MSCs, IFNγ+ UC-MSCs, and IFN-γ+ TPL-primed UC-MSCs versus UC-MSCs as control. The values are shown by log2 ratio. [file Table_2.pdf]

Table 2.

The gene expressions of HLA-A, HLA-B, and HLA-C in UC-MSCs by microarray analysis

Gene Symbol	TPL-primed UC-MSC to UC-MSC (No33 lot)	IFN-γ-primed UC-MSC to UC-MSC (No33)	IFN-γ and TPL-primed UC-MSC to UC-MSC(No33)	TPL-primed UC-MSC to UC-MSC (No35 lot)	IFN-γ-primed UC-MSC to UC-MSC (No35)	IFN-γ and TPL-primed UC-MSC to UC-MSC(No35)	
	SET01 Log2ratio	SET02 Log2ratio	SET03 Log2ratio	SET04 Log2ratio	SET05 Log2ratio	SET06 Log2ratio	Probe Set ID
HLA-A	-0.28363	3.82366	3.198158	-1.04817	4.031767	2.688925	TC0600007495.hg.1
HLA-B	0.696424	4.998448	4.458509	-0.62117	4.512793	4.090449	TC0600014258.hg.1
HLA-C	0.041696	3.792738	3.291949	-1.51609	3.934149	2.963895	TC0600014257.hg.1
